# Nitrites Detection with Sensors Processed via Matrix-Assisted Pulsed Laser Evaporation

**DOI:** 10.3390/nano12071138

**Published:** 2022-03-29

**Authors:** Cristina Craciun, Florin Andrei, Anca Bonciu, Simona Brajnicov, Tatiana Tozar, Mihaela Filipescu, Alexandra Palla-Papavlu, Maria Dinescu

**Affiliations:** 1 Lasers Department, National Institute for Laser, Plasma and Radiation Physics, 077125 Magurele, Romania; cristina.craciun@inflpr.ro (C.C.); florin.andrei@inflpr.ro (F.A.); anca.bonciu@inflpr.ro (A.B.); brajnicov.simona@inflpr.ro (S.B.); tatiana.alexandru@inflpr.ro (T.T.); mihaela.filipescu@inflpr.ro (M.F.); maria.dinescu@inflpr.ro (M.D.); 2 Faculty of Physics, University of Bucharest, 077125 Magurele, Romania; 3 Faculty of Chemistry, University of Bucharest, 030018 Bucharest, Romania

**Keywords:** chitosan, carbon nanotubes, composites, MAPLE, electrochemical sensor, nitrite, LOD

## Abstract

This work is focused on the application of a laser-based technique, i.e., matrix-assisted pulsed laser evaporation (MAPLE) for the development of electrochemical sensors aimed at the detection of nitrites in water. Commercial carbon-based screen-printed electrodes were modified by MAPLE via the application of a newly developed composite coating with different concentrations of carbon nanotubes (CNTs), chitosan, and iron (II) phthalocyanine (C_32_H_16_FeN_8_). The performance of the newly fabricated composite coatings was evaluated both by investigating the morphology and surface chemistry of the coating, and by determining the electro-catalytic oxidation properties of nitrite with bare and modified commercial carbon-based screen-printed electrode. It was found that the combined effect of CNTs with chitosan and C_32_H_16_FeN_8_ significantly improves the electrochemical response towards the oxidation of nitrite. In addition, the MAPLE modified screen-printed electrodes have a limit of detection of 0.12 µM, which make them extremely useful for the detection of nitrite traces.

## 1. Introduction

Nitrites (compounds that contain the nitrite ion NO_2_^−^) are widely studied for their applications in food and chemical industries, but also for their potential toxicity. In particular, the detection of nitrites is important due to their potential to affect human health leading to liver damage, methemoglobinemia, and even cancers [1]. In addition, nitrites cause methemoglobinemia by oxidizing the Fe^2+^ of haemoglobin [2]. Furthermore, according to the World Health Organization, the nitrate concentration in drinkable water should not exceed 65.2 µM (3 ppm) [3].

In order to monitor the presence of nitrites in water, food, and environmental systems, many detection methods are designed. Colorimetry and spectrophotometry are some early detection techniques, but these methods have low accuracy and are easily affected by other substances. A technique with better precision but with high cost and poor portability is gas/liquid chromatography-mass spectrometry [4]

Therefore, in recent years, electrochemical sensors have been intensely used as an alternative for detecting nitrites in water, due to their low cost and high portability. The primary mechanism for detection is based on the electro-oxidation of nitrite ions, which produces a modification in the signal recorded by the sensor. Generally, the oxidation rate depends on the electron transfer kinetics and electro-active ability of the electrode. The most common materials used for the fabrication of electrodes are platinum, gold, copper, silver, carbon, and glassy carbon. The central performances desired from an electrochemical sensor are the linearity of the sensor’s output with the recorded signal, a low limit of detection (LOD), high selectivity for a specific contaminant, and high sensitivity. The modification of the electrode with carbon nanomaterials, such as carbon nanotubes (CNTs), metallic nanoparticles (NPs), macromolecules, and polymers, can improve the detection performances of the electrochemical sensors [4,5,6,7].

Few examples electrochemical sensors based on carbon nanotubes doped or decorated with different nanoparticles are reported in [8,9,10,11]. The authors use the sensors for the detection of nitrites and they report LOD between 0.001 and 0.5 µM.

An interesting organic material studied for biosensors is chitosan, which is generally combined with metal-oxide nanoparticles or carbon nanotubes to increase its electrical properties for sensing applications [12,13]. Bibi et al. [14] presented an MWCNT paste electrode modified with chitosan functionalized Ag-NPs while Li et al. [15] reported a sensor based on chitosan, MWCNTs, and carbon nanoparticles (CNs), and Bai et al. presented a multilayer film sensor based on H_7_P_2_Mo_17_V_1_O_62_ (P_2_Mo_17_V)-carbon nanotubes and Pt-chitosan [16].

A novel highly sensitive electrochemical sensor is reported by Wang et al. [17]. A Chit/Ti_3_C_3_ solution was deposited on glassy carbon electrodes, and the obtained electrode is immersed in a phosphate buffer solution containing HAuCl_4_ and reduced by cyclic voltammetry in order to deposit gold nanoparticles. The resulted electrode AuNPs/Chit/Ti_3_C_2_T_x_/GCE showed good performance, two linear ranges, 0.5–335 μM and 335–3355 μM, and LOD of 0.069 μM. In their previous research [18], a AuNPs/Ti_3_C_2_T_x_/ERGO/GCE sensor was designed exhibiting linear ranges 0.5–80 μM and 80–780 μM with limits of detection 0.15 μM and 0.051 μM for nitrites sensing.

Furthermore, biological compounds are studied for sensor applications. For instance, a high-performance electrochemical sensor based on SWCNTs covalently immobilized with single-strand deoxyribonucleic acid (SWCNTs-ssDNA) for the detection of nitrite is presented by Xian et al. [19], while in Yang et al. [20], DNA functionalized single-wall carbon nanotubes/Cu^2+^ (DNA–CNTs/Cu^2+^) are shown. The Cu^2+^ modified SWCNTs-ssDNA sensor exhibited lower LOD and a more extensive linear range.

Thus, in this work we aim at the application of a laser-based deposition approach, i.e., matrix-assisted pulsed laser evaporation (MAPLE) [21,22,23] for the development of an electrochemical sensor. Briefly, the MAPLE process is similar to the conventional pulsed laser deposition (PLD) method which involves the interaction of a laser beam with a solid target inside of a vacuum chamber and the formation of a plasma plume which condenses onto a substrate to form a thin film. The main difference between the two techniques is the solid target, i.e., in MAPLE, the sensitive material is suspended in a solvent at low concentrations (also called matrix) and flash frozen in liquid nitrogen. The most important advantage of MAPLE is its high flexibility, allowing most of the materials which can be dissolved or suspended in a highly volatile and light-absorbing solvent to be deposited as a thin film [22,23]. Other advantages are related to: (i) a better control of the film thickness and surface morphology, (ii) an enhanced film/substrate adhesion, (iii) the usage of low quantities of material, and (iv) it is compatible with low laser fluences.

In this work, an electrochemical sensor based on a carbon nanotube (CNTs), chitosan, and iron (II) phtalocyanine (C_32_H_16_FeN_8_) (abbreviated CNT-Chit-FePc) composite is reported. The CNT-Chit-FePc composite is applied as a thin film by matrix-assisted pulsed laser evaporation (MAPLE) onto the surface of commercially available carbon-based screen-printed electrodes (C-SPE). The novelty and advantage of this work arises from the possibility to apply a facile one-step strategy, i.e., MAPLE, to obtain hybrid coatings with promising performance in nitrite sensing. In addition, the laser-processed C-SPEs exhibit satisfactory selectivity and stability, demonstrating its capability and promise in trace nitrite determination in water. The carbon-screen printed electrode modified by MAPLE with a CNT-Chit-FePc composite, by virtue of its simplicity, ease of fabrication and use, could be extended for detection of nitrites in the human body. For example, C_32_H_16_FeN_8_ has a similar structure to haemoglobin [24], and thus Fe^3+^ reduces NO_2_^−^ ions [25].

## 2. Materials and Methods

### 2.1. Laser Processing of the Sensing Materials for the Working Electrode

The electrochemical sensor sensitive layer was based on a composite containing CNT (carbon nanotube, single-walled, solvent-based conductive ink from Merck KGaA, Darmstadt, Germany), chitosan (natural polymer from Merck KGaA, Darmstadt, Germany), and iron (II) phthalocyanine (from Merck KGaA, Darmstadt, Germany) with improved physical and chemical properties, due to their complementary behaviour that is not possible from their single counterparts.

**Figure 1:**
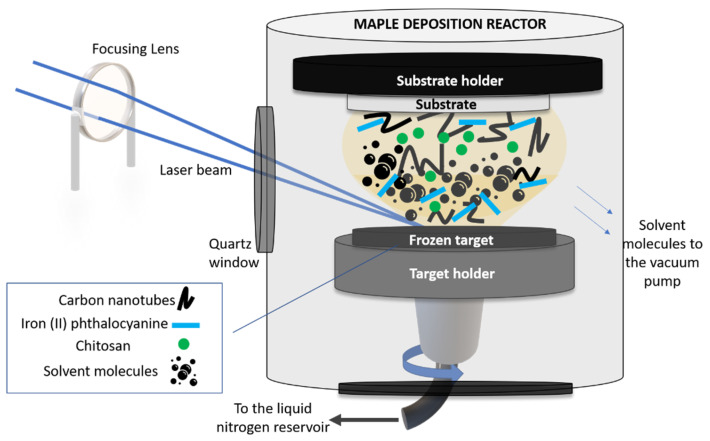
Sketch of the MAPLE setup used in this work.

The sensitive layer was applied on the working electrode of a commercial carbon-based screen-printed electrode (C-SPE) (from Dropsense, Asturias, Spain) by means of matrix-assisted pulsed laser evaporation (MAPLE) (commercial system from Neocera LLC, Beltsville, MD, USA). A sketch of the MAPLE experimental setup used in this work is shown in Figure 1.

In order to carry out the MAPLE experiments, we first fabricated the solid targets which were irradiated by the laser beam. In this work, the CNT-Chit-FePc mixture was suspended in water (at different concentrations, see Table 1) and flash frozen using liquid nitrogen, resulting a solid target. The choice of the different concentrations used in this work was based on our preliminary study reported in [26], where we proved the possibility to process new composites based on (CNT (2%), chitosan (0.5%), and C_32_H_16_FeN_8_ (0.4%) without structural modifications. Chitosan was used as a matrix for the entrapment of the composite based on CNT and C_32_H_16_FeN_8_ and allowed for a uniform and controllable distribution of the compounds in the coating. Moreover, it was also found that the impact of FePc loading becomes saturated for high loadings (i.e., from 50 to 100 mg) [27].

The frozen solid target was then inserted into the vacuum chamber (working pressure of 5 × 10^−5^ mbar) and irradiated with a pulsed Nd:YAG laser beam (Surelite II from Continuum, Bordeaux, France) (λ = 266 nm, ν = 10 Hz and τ = 5–7 ns). The laser beam was guided and focused through an optical system (mirrors and lens) in the processing chamber, at an angle of incidence of ~45° on the surface of the solid target. The laser fluence was set at 580 mJ/cm^2^.

Upon irradiation, the solvent evaporated and was pumped out of the deposition chamber, allowing only for the composite material to be collected as a thin film on a substrate placed parallel to the target at a distance of approximately 4 cm. The target was rotated by a motor and at the same time the laser beam was scanned onto the surface of the target to achieve a uniform evaporation. The substrates used to collect the thin film materials were Si(100) (used for post-deposition morphological and chemical investigations) and C110 sensors from Metrohm Dropsens, Asturias, Spain (C-SPE). The thickness of the as-deposited layers was approx. 80 nm (please see Figure S1).

### 2.2. Characterization of the Sensing Coatings Processed by MAPLE

The morphological features of the laser processed sensing layers were investigated by FEI Inspect-S scanning electron microscope (Thermo Fisher Scientific Inc., Waltham, MA, USA) at an accelerating voltage between 5–20 kV.

Fourier-transform infra-red (FTIR) spectroscopy was used to obtain the infra-red (IR) absorption spectra of the as-deposited coatings. The IR spectra of the coatings were recorded as a mean of 16 spectra with a Nicolet FT-IR iS50 spectrometer (Thermo Fisher Scientific, Waltham, MA, USA) equipped with an attenuated total reflection (ATR) module. The resolution was 4 cm^−1^ and the spectral range 4000–650 cm^−1^. FTIR-ATR measurements were carried out using a ZnSe crystal with the following properties: 1.5 mm diameter, one internal reflection at a 42° angle of incidence, and 2.03 µm depth of penetration at 1000 cm^−1^.

The surface wettability of the sensing layers was measured by using the sessile drop method applied at constant temperature of 20 °C. A droplet (4 μL) was placed on the surface of the as-deposited coatings and the contact angle measurements were performed using a KSVCAM101 microscope (KSV Instruments Ltd., Espoo, Finland) equipped with a video-camera and FireWire interface, which allowed the acquisition of images with a resolution of 640 × 480 pixels. The surface free energy (SFE) was calculated using two wet agents: deionized water as a polar liquid, and for the completely dispersive liquid, di-iodomethane. The final SFE values were extracted from contact angle measurements and conducted using the concept of polar and dispersion components by means of the Owens, Wendt, Rabel, and Kaelble (OWRK) method for estimation [28,29,30].

For the electrochemical investigation of the sensors based on CNT-Chit-FePc, all chemicals were analytical grade and used with no further purification. Sodium nitrite (NaNO_2_—Merck KGaA, Darmstadt, Germany, purity ≥ 99.0%) was used for the preparation of solutions containing nitrite ions. The standard Briton Robinson (BR) buffer was prepared by using acetic acid (CH_3_COOH—Sigma-Aldrich, purity ≥ 99%), boric acid (H_3_BO_3_—Sigma-Aldrich, purity ≥ 99.5%), and phosphoric acid (H_3_PO_3_—Merck KGaA, Darmstadt, Germany). The electrochemical measurements were performed using an AutoLab PGSTAT302N (Utrecht, The Netherlands) controlled by NOVA 1.11 software (Utrecht, The Netherlands). Detection of nitrite was studied using both voltammetric and amperometric techniques. All samples were tested in a 10 mL electrochemical reactor, filled before each measurement with fresh solution of 0.1 M BR electrolyte. During the voltammetric investigation of sensors, the potential was varied from 0.1 V to 1.2 V (vs. Ag/AgCl), and vice versa. The pH value of the supporting electrolyte was adjusted between 3 and 7 and its influence on the electrochemical process was rigorously studied. Nitrite oxidation was amperometrically studied at an applied potential of 0.8 V vs. Ag/AgCl in the same electrochemical cell. Nitrite content was added step-by-step to the electrolyte solution using the standard addition method.

## 3. Results and Discussions

### 3.1. Surface Chemistry Investigations: FTIR, Contact Angle, and Surface Free Measurements

The successful laser deposition of the composite coating, i.e., CNT-Chit-FePc, and the investigation of the surface functional groups was evaluated by Fourier transformed infra-red (FTIR) spectroscopy.

First, the IR spectra of the starting materials, i.e., chitosan, C_32_H_16_FeN_8_, and CNTs pre-processed by MAPLE, were investigated. The acquired spectra of the starting materials, shown in Figure S2a–c, are similar to those reported in [31,32,33,34,35,36].

**Figure 2:**
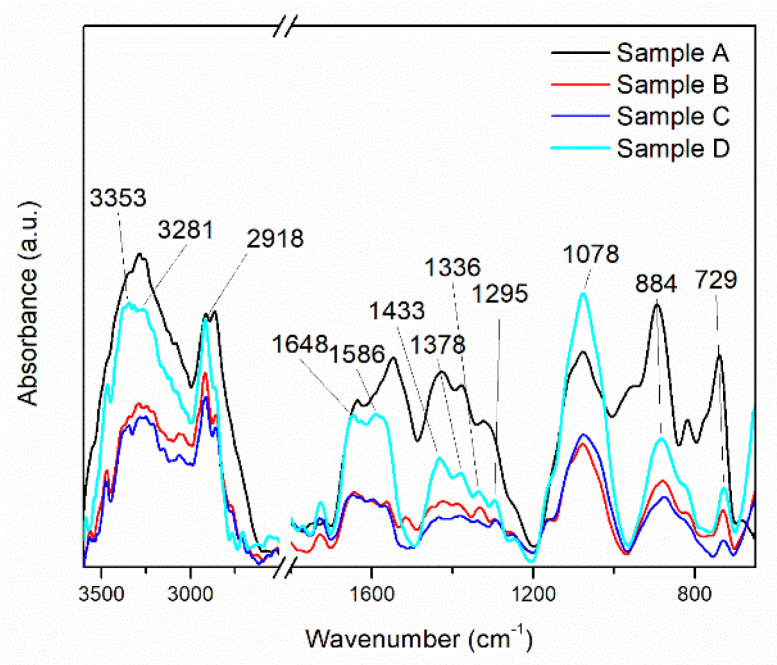
This FT−IR spectra of the MAPLE deposited CNT-Chit-FePc composite from different targets: Sample A (target containing (15% CNT + 1% Chit + 1% C_32_H_16_FeN_8_) in H_2_O); Sample B (target containing (15% CNT + 1% Chit + 0.4% C_32_H_16_FeN_8_) in H_2_O); Sample C (target containing (22% CNT + 1% Chit + 0.4% C_32_H_16_FeN_8_) in H_2_O); Sample D (target containing (22% CNT + 1% Chit + 1% C_32_H_16_FeN_8_) in H_2_O).

In the FTIR spectra of the MAPLE-deposited CNT-Chit-FePc composites (shown in Figure 2), chitosan is identified by the bands at 1648 cm^−1^ (C=O stretching vibration of amide I), 1586 cm^−1^ (N–H bending vibration of amide II), 1378 cm^−1^ (C–H symmetrical deformation vibrations of CH_3_), 1078 cm^−1^ (C–O stretching vibration), and 884 cm^−1^ (C–H bending out of the plane of the ring of monosaccharides). Some of the bands are shifted when compared with the bands of individual chitosan. C_32_H_16_FeN_8_ is recognized in the thin films at 1336 cm^−1^ (C–N stretching vibration) and 729 cm^−1^ (C–N bending vibration), whereas CNT at 1245 cm^−1^ and 1295 cm^−1^ (C–H bending vibrations and combinations of OH deformation and of C–O stretching vibrations).

Despite CNT having the highest ration content of all the materials, the existence of only two bands in the CNT-Chit-FePc composite IR spectrum is due to CNT’s weak dynamic dipole moment when compared with that of chitosan and C_32_H_16_FeN_8_ [35]. The disappearance from the CNT-Chit-FePc composite IR spectrum of the CNT bands at 1609, 1510, 1453, 1362, 1348, 1185, and 1103 cm^−1^ indicates that the functional groups on the surface of the CNT were consumed or neutralized during the composite formation process. Furthermore, electrostatic interaction between chitosan and CNT could explain the shifted bands observed for chitosan in the CNT-Chit-FePc composite IR spectrum when compared to individual chitosan [35]. The successful deposition of the starting materials as thin films without affecting their structural damage can be confirmed by the presence of their characteristic IR bands without any significant changes in peak positions in the CNT-Chit-FePc composite IR spectrum.

The successful deposition of the starting materials as thin films without affecting their structural damage can be confirmed by the presence of their characteristic IR bands without any significant changes in peak positions in the CNT-Chit-FePc composite IR spectrum. Therefore, MAPLE is eligible for composite transfer from the frozen matrix to the collector as a thin layer without noticeable chemical/structural damages.

**Figure 3:**
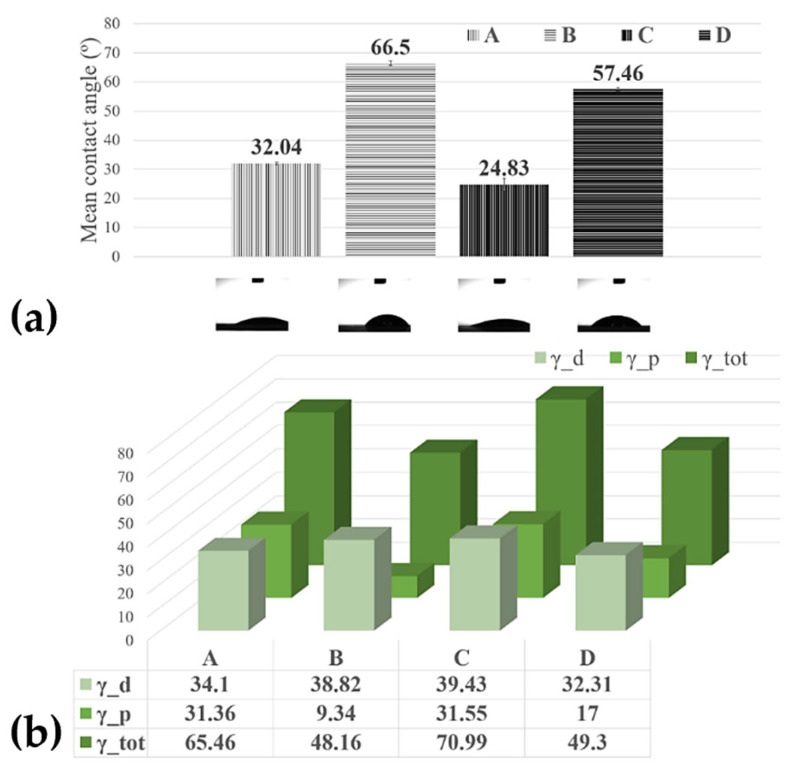
(**a**) Histograms of the contact angles measured on the MAPLE processed surfaces; (**b**) surface free energy of the MAPLE processed surfaces from targets with the different compositions: Sample A (target containing (15% CNT + 1% Chit + 1% C_32_H_16_FeN_8_) in H_2_O); Sample B (target containing (15% CNT + 1% Chit + 0.4% C_32_H_16_FeN_8_) in H_2_O); Sample C (target containing (22% CNT + 1% Chit + 0.4% C_32_H_16_FeN_8_) in H_2_O); Sample D (target containing (22% CNT + 1% Chit + 1% C_32_H_16_FeN_8_) in H_2_O).

Contact-angle measurements were used on a large scale for the characterization of thin film surface wettability, and the results are presented in Figure 3.

The contact angle of water at the surface of the bare C-SPE was measured, and the value was ~73°. However, it decreased after the C-SPE was coated with CNT-Chit-FePc. This increase in the hydrophilicity of the coated electrode indicates that the properties of the CNT-Chit-FePc composite can be tuned using a laser evaporation technique. In the study reported in [37] it was found that the ammonia group in the chitosan molecular chain has a strong affinity to the CNT surface, thus potentially explaining the higher contact angles of the sample D (57.46°) compared with sample A (32.04°), and sample B (66.5°) compared with the sample C (24.83°), respectively. Some theoretical calculations prove the helical wrapping on CNTs is the optimal configuration for polymers of rigid molecular chains [38,39]. The biopolymer chitosan is helically wrapped on the CNT surface, favouring CNT solubilization in water. A recent experiment revealed that the absorption of biopolymer chitosan on the CNT surface is influenced by the deacetylation degree of the chitosan molecular chain [40]. A low deacetylation degree provides more hydrophobic sections that favour the absorption of chitosan on the CNT surface, while a high deacetylation degree provides a higher electrostatic repulsive force. The high contact angle (96.20°) of the pure chitosan film may be attributed to the hydrophobic backbone of chitosan chains [41]; therefore, the coatings where the chitosan wets the CNT and C_32_H_16_FeN_8_ well, i.e., samples D (57.46°) and B (66.5°), a higher contact angle is expected (Figure 3). When the film presents C_32_H_16_FeN_8_ protrusions from the surface, such as in the modified C-SPE with sample A (32.04°) and sample C modified C-SPE (24.83°), the water contact angle decreases, likely due to the hydroxyl groups from the Fe species [42]. This observation confirms that the hydrophilicity and wettability of the composite coating can be improved by incorporating C_32_H_16_FeN_8_ into the polymeric matrix in the fabrication process.

As previously mentioned, allowing the design and development of surfaces that interact in a specific way to promote desired processes and minimize detrimental side effects is the key to a successful electrochemical sensor. When fluids come in contact with artificial materials, water interactions and liquid adsorption are governed by the surface free energy of the material. Polymers are often considered low-energy surfaces due to their covalent and Van der Waals bonding, therefore often leading to the surfaces being non-polar and thus of a hydrophobic nature. In our case, using a laser evaporation technique, we obtained, for the most hydrophilic probe, the C-SPE modified with Sample B, and a surface free energy of 70.99 mN/m with the highest dispersive component of 39.43 mN/m (Figure 3), demonstrating the ability to maintain the viability and functionality of composite’s compounds in aqueous media.

### 3.2. Morphological Investigations

**Figure 4:**
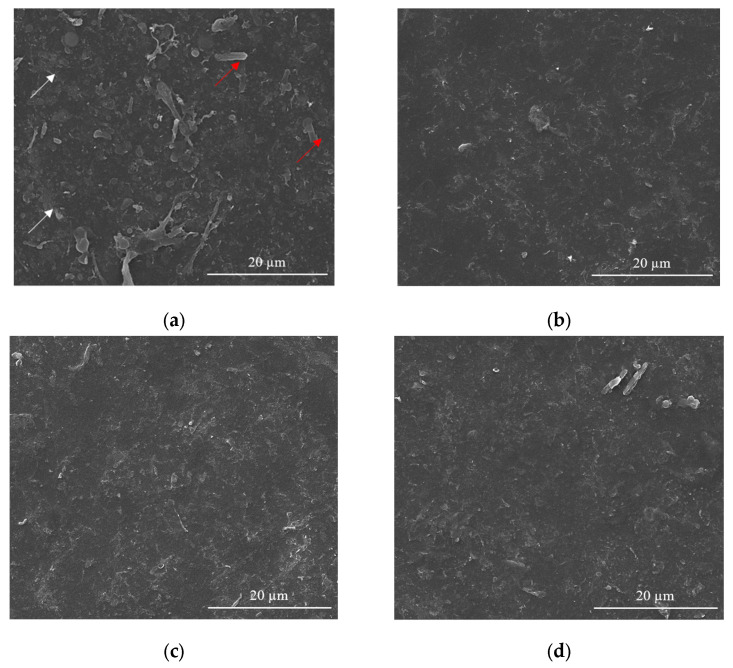
SEM images for of the CNT-Chit-FePc coatings obtained on Si (100) by MAPLE from targets with different compositions: (**a**) (15% CNT + 1% Chit + 1% FePc) in H_2_O (sample A); (**b**) (15% CNT + 1% Chit + 0.4% FePc) in H_2_O (sample B); (**c**) (22% CNT + 1% Chit + 0.4% FePc) in H_2_O (sample C); and (**d**) (22% CNT + 1% Chit + 1% FePc) in H_2_O (sample D).

Usually, in the sensing process, as most devices require homogeneous coatings over large surfaces (over 100 µm^2^), MAPLE is the method of choice among the various laser deposition methods [43]. Therefore, SEM investigations carried out on CNT-Chit-FePc coatings deposited on Si(100) substrates reveal uniform coatings with different features, such as “worm”-like (white arrows in Figure 4a) and rods (red arrows in Figure 4a) that can be potentially assigned to CNT bundles and to iron phthalocyanine, respectively (please see also the Supplementary Materials for atomic force microscopy images of the features observed in SEM, i.e., Figure S3). Generally, the micrometric aggregation of composite is uniformly distributed, leading to a high specific surface area of the layers that is ideal for gas molecules adsorption [44]. For a qualitative analysis of the as-deposited layers (EDX investigation), please see Figure S4 in Supplementary Materials, where it can be seen that all the coatings are uniformly formed on the substrate.

Samples (A and B) obtained from targets with a lower concentration of CNT (i.e., 15 wt %t) have surfaces with composite material splashes (Figure 4a,b); meanwhile, a higher CNT concentration in the target, i.e., 22 wt %, leads to a better uniformity of the layer (samples C and D) (please see Figure S5 for higher magnification images). The CNTs are uniformly scattered and embedded in the composite matrix (Figure 4c,d). The films appear to be continuous, homogenous, and without voids. This may indicate that when the CNTs, C_32_H_16_FeN_8_, and chitosan arrive at the sample surface, chitosan is still in the liquid state, and fill voids in the film before solidification.

The film is free of CNT protrusions from the surface, as in Figure 4a, indicating that the chitosan wets the CNT and C_32_H_16_FeN_8_ well in this case. The structure of the film shown in Figure 4a indicates that the growth mode is different to the films shown in Figure 4b–d. We hypothesize that the CNTs and C_32_H_16_FeN_8_ are “structurally reinforced” by the chitosan when they arrive at the surface, and act as nucleation and growth sites for the chitosan during the transfer process. When the CNT and C_32_H_16_FeN_8_ arrive on the sample surface, they are coated with chitosan, and they preserve their orientation and shape. Chitosan that is transferred subsequently and that does not encounter CNTs or C_32_H_16_FeN_8_ will be deposited on the substrate forming the observed chitosan film underneath. This influence of the growth mode on the morphology of the film is also explored by Wu et al. [45].

Chitosan is used as a matrix for the entrapment of a composite based on CNT and C_32_H_16_FeN_8_ onto C-SPE platform to fabricate an electrochemical sensor. High concentration of CNTs single dispersion and stabilization could be achieved by varieties of polysaccharides such as chitosan [46].

**Figure 5:**
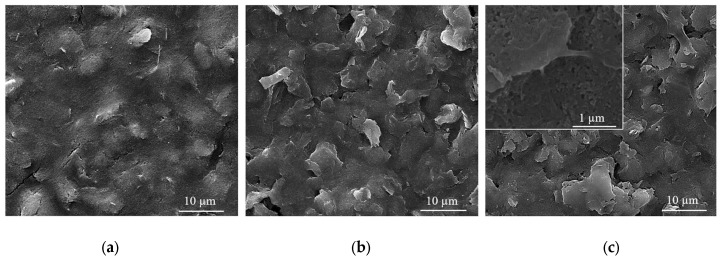
SEM images of (**a**) bare C-SPE surface; (**b**) C-SPE coated with a CNT-Chit-FePc film obtained from a (22% CNT + 1% Chit + 0.4% FePc) in H_2_O target; (**c**) the same C-SPE coated with a CNT-Chit-FePc film after 200 cyclic voltammograms.

A C-SPE coated by MAPLE with a CNT-Chit-FePc film (sample C) after 200 cyclic voltammograms in the standard BR buffer at pH 4 is shown in Figure 5. The CNTs and C_32_H_16_FeN_8_ largely preserve the orientation and shape they acquired after deposition, while the chitosan matrix presents a non-uniform coating due to the long exposure in the BR buffer. The need to use a polymeric matrix arises from the long-term electrochemical assays and use, as well as from the necessity to have a controllable and uniform distribution of the compounds as a coating on the device, demonstrating the ability of its use to maintain the functionality of the composite compounds.

In 2007, Wang et al. investigated the solution behaviour of chitosan-wrapped CNTs [46]. The comparative characterization indicates that the electrostatic repulsive force of the charged chitosan and its derivate molecular chains could stabilize their wrapped CNTs. The aggregation of chitosan and its derivate-wrapped CNTs happened when they were discharged by changing the pH value of their suspension. When the pH value of suspension for chitosan-wrapped CNTs is higher than 6.59, the -NH_3_^+^ group deprotonated into -NH_2_, and a precipitate could be observed. The CNTs functionalized by chitosan derivates that contain COOH group deprotonate in an acidic environment of pH lower than 4.66 aggregate [47]. This could explain the micrometric aggregation of composite shown in Figure 5 after long exposure in the acid buffer at pH 4.0.

To sum up, we have shown that MAPLE is an efficient method to obtain composite coatings, allowing the design and development of surfaces that interact in a specific way to promote the desired processes and minimize detrimental side effects [43,48].

### 3.3. Voltammetric Response of Nitrite at CNT-Chit-FePc Modified C-SPE

Literature studies on electrochemical sensors based on MAPLE-deposited coatings lack important data on various important characteristics such as reproducibility, interferences, operational and storage stability, and the influence of experimental parameters such as pH, being thus far from applications in real samples.

**Figure 6:**
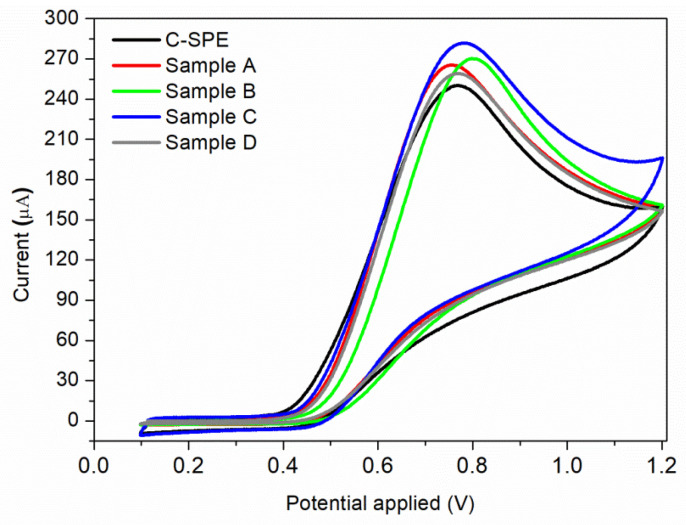
Cyclic voltammograms of 7 × 10^−3^ M nitrite solution using a commercial C-SPE sensor, and sensors modified by MAPLE under different conditions: Sample A; Sample B; Sample C; Sample D.

The electrocatalytic oxidation properties of nitrite with bare and MAPLE-modified commercial carbon-based screen-printed electrodes (C-SPE) were investigated. Each sensor was immersed into the electrochemical cell which was filled with the electrolyte solution having the desired nitrite content. The recorded voltammograms in BR buffer (pH = 4.0) containing 7 × 10^−3^ M nitrite, at a scan rate of 0.05 V/s, obtained for the unmodified C-SPE and samples A–D by MAPLE under different conditions, are presented in Figure 6. For all samples, only the anodic peak is present, with no cathodic peak being observed during the reverse scan, meaning that nitrite ions are irreversibly oxidized to nitrate. This effect has been found previously [8,49], where the electrochemical irreversibility behaviour of nitrite was confirmed. Modifying a simple C-SPE via MAPLE by applying a thin nanostructured coating leads to an overall improvement of the measured current, all samples showing higher values than the initial sensor. However, the highest value of the current (281 μA) is obtained for Sample C at an applied potential of 0.78 V vs. Ag/AgCl. This can be correlated to its high CNTs composition, which leads to a complex sensor having high specific surface area. Considering that the highest current was obtained for sample C, further investigations are focused on this sensor.

The reproducibility of the modified C-SPE (with Sample C) for the determination of nitrite was evaluated by carrying out 10 voltammetric cycles in a buffer solution containing 7 × 10^−3^ M nitrite. The relative standard deviation (RSD) for the electrode response was 3%, which means that the reproducibility of the sensor is in the acceptable range.

**Figure 7:**
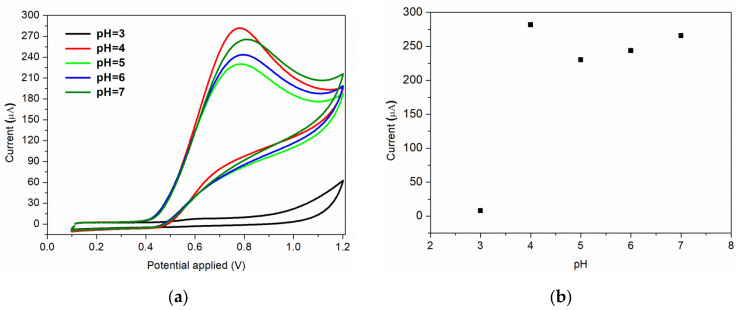
(**a**) Effect of pH of electrolyte on the voltammetric response; (**b**) corresponding plot of peak current vs. pH.

Furthermore, the nature and the pH of the electrolyte solution have a major impact on the electrochemical processes. The supporting electrolyte for this study was Briton Robinson buffer, with a pH range in acidic medium from 3 to 7. This electrolyte was chosen because it has been demonstrated previously that in BR buffer, a more sensitive and sharper anodic peak for nitrite oxidation is obtained, compared to the peak recorded in other supporting electrolytes [49]. The voltammograms recorded for 7 × 10^−3^ M nitrite, as a function of pH values, are shown in Figure 7a. As it can be observed, a very small oxidation peak is obtained when an electrolyte solution of pH = 3 is used. A possible explanation of this behaviour can be correlated to the protonation of nitrite ions or to the conversion of NO_2_ to NO at small pH values [8]. The highest anodic peak is obtained for pH = 4, which is the pH value used for further studies. The same result is obtained by S. Bibi et al., the authors mentioning that at pH = 4 the nitrite is electrochemically accumulated on the chitosan-CNW sensor’s surface, due to the amino and hydroxyl groups charging [14]. A relative linear increase in the anodic current is observed for pH range of 5–7, as can be seen in Figure 7b.

**Figure 8:**
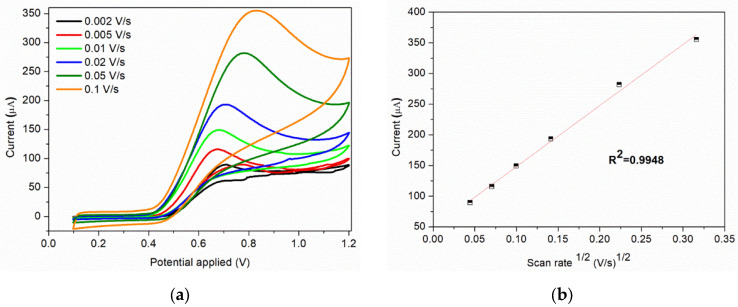
(**a**) Cyclic voltammograms of 7 × 10^−3^ M nitrite solution at various scan rates; (**b**) Corresponding plot of peak current vs. square root of scan rate at 7 × 10^−3^ M nitrite.

The scan rate is an important parameter that can influence the current response of nitrite. The cyclic voltammograms of sample C at different scan rates obtained in 0.1 M BR buffer (pH = 4) containing 7 × 10^−3^ M nitrite are show in Figure 8a. The anodic peak current increases with the scan rate (0.002–0.1 V/s), this increase being assisted by a shift of the peak toward more positive potentials and broadening of the peak. The peak current obtained for the anodic oxidation of nitrite ions is proportional to the square root of scan rate (Figure 8b), indicating that the process is controlled by diffusion.

**Figure 9:**
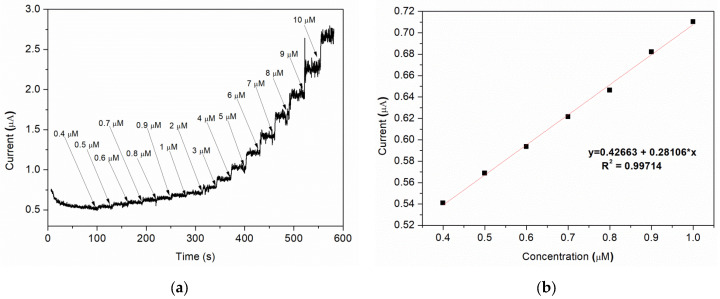
(**a**) Amperometric response of various nitrite concentration; (**b**) corresponding plot of peak current vs. nitrite concentration (linear calibration plot).

Amperometric studies were used to analyse the current response for each addition of nitrite under hydrodynamic condition. A typically steady-state amperometric response of Sample C with a successive injection of nitrite, under constant stirring, into a 0.1 M BR buffer (pH = 4) solution at an applied potential of 0.8 V vs. Ag/AgCl is presented in Figure 9a. Different volumes of buffer solution containing a specific amount of nitrite are added at 30 s intervals, the final concentration range of nitrite being 0.4–10 μM. As it can be observed, for each addition of nitrite, the current rapidly increases and the steady-state value is reached after ca. 3–4 s. A good linear correlation between the current and the nitrite concentration is obtained for 0.4–1 μM range, with a regression coefficient (R^2^) = 0.997 (Figure 9b).

The limit of detection (LOD) is found to be 0.12 μM using the formula [50]: LOD = 3S_a_/b, where “S_a_” is the standard deviation of the response and “b” is the slope of the calibration curve.

A comparison between different sensors tested in buffer solutions with different pH and their limit of detection is shown in Table 2. As it can be seen, in our case, good results are obtain using the modified MAPLE sensors proving that this technique is suitable for processing new functionalized composites as thin layers.

**Figure 10:**
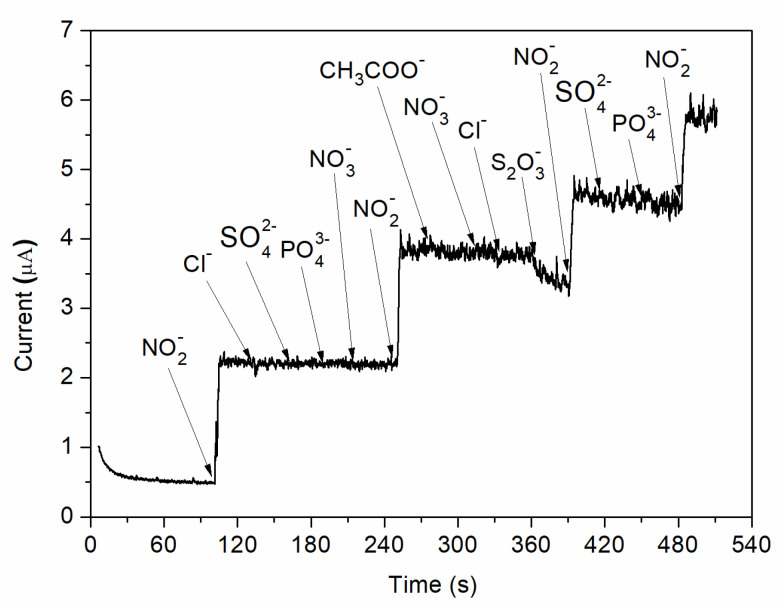
Amperometric response of nitrite and various possible interfering species.

In addition, the effect of interfering species was analysed by adding different possible inferring substances in 0.1 M BR buffer (pH = 4) solution at an applied potential of 0.8 V vs. Ag/AgCl. As can be observed in Figure 10, no visible increase in the measured currents is observed when chosen ions (Cl^−^, SO_4_^2−^, PO_4_^3−^, NO_3_^−^, CH_3_COO^−^, S_2_O_3_^2−^) are added into the electrochemical system. However, a strong increase in the amperometric current is observed when the same concentration of nitrite is injected. Therefore, the C-SPE sensor with the surface modified by MAPLE, i.e., with a carbon nanotubes–chitosan–phthalocyanine composite, shows a good selectivity for nitrite concentration.

Finally, practical applications of the MAPLE processed C-SPEs were envisioned and the nitrite level in water from a well was evaluated. The real samples were used without further purification or separation procedures. Nitrite content was amperometrically detected using the same standard addition method described above. The determination results are resumed in Table 3. As can be observed, a relatively high content of nitrite is found in the analysed water. Good percentage recoveries in the range of 98–115% are obtained, meaning that nitrites can be accurately determined in real samples.

## 4. Conclusions

The main challenge in the field of electrochemical sensors is the development and application of multifunctional materials with specific properties or functions. Matrix-assisted pulsed laser evaporation (MAPLE) offers unique advantages in obtaining composite coatings without noticeable chemical/structural damages on any substrate. In this work, we applied MAPLE for the fabrication of new composite coatings based on carbon nanotubes (CNTs), chitosan (Chit), and iron (II) phthalocyanine (FePc) which were deposited as thin films onto commercial carbon-based screen-printed electrodes (C-SPE). The goal of the work was to demonstrate the potential of the newly developed C-SPE for the detection of low concentrations of nitrites in water.

Following MAPLE, we obtained CNT-Chit-FePC homogeneous composite coatings, with different “worm”- and “rod”-like features that can be assigned to CNT bundles and to iron phthalocyanine (FePc). The MAPLE processed composite was uniformly distributed onto the C-SPEs, leading to a high specific surface area of the layers, making it ideal for gas molecules adsorption.

In addition, the C-SPE’s modified by MAPLE were employed for the voltammetric determination of nitrite. The sensor measurements were carried out in a Briton Robinson buffer solution at different pH values. Different nitrite concentrations were added into the electrolyte solution, and, by applying an external potential, the nitrite ions (NO_2_^−^) were oxidized, at the sensor surface, to nitrate ions (NO_3_^−^). The oxidation peak appears at 0.8 V vs. Ag/AgCl. This potential was used for further amperometric detection of nitrite ions, where it can be observed that for each addition of nitrite content, the generated current increases. We assume that the combined effect of CNT, chitosan, and iron (II) phthalocyanine improves the electrochemical response towards the oxidation of nitrite. This is emphasized by the ability of CNT-Chit-FePc composite produced by MAPLE to adsorb rapidly the nitrite at the surface leading to the increase in sensitivity towards nitrite.

Moreover, the C-SPE fabricated from the starting target 22% CNT + 1% Chit + 0.4% C_32_H_16_FeN_8_ (Sample C) shows the highest anodic current for nitrite oxidation. The limit of detection for this sensor is 0.12 µM, meaning that the resulting sensor can be successfully used even for the detection of nitrite traces. The same electrode has an excellent selectivity, toward nitrite ions in comparison to other common anions, none of them showing an amperometric response. In addition, the C-SPE can be successfully used for real sample analysis.

To sum up, this work shows an important direction for the development of nitrite detection sensors based on novel hybrid composites (i.e., carbon nanotubes, chitosan, and iron (II) phthalocyanine) fabricated by MAPLE. Practical application aspects of the proposed sensor include detection of nitrites in the food industry or in medical applications. There are additional questions for future research, such as whether we can detect nitrites in other environments (i.e., blood) or how the sensor performances would depend on the topography and surface chemistry/wettability of the MAPLE processed composite coatings.

## Figures and Tables

**Table 1:** Composition of the targets used for the MAPLE experiments.

Sample No.	Target Composition (wt %) in H_2_O
A	15% CNT + 1% Chit + 1% C_32_H_16_FeN_8_
B	15% CNT + 1% Chit + 0.4% C_32_H_16_FeN_8_
C	22% CNT + 1% Chit + 0.4% C_32_H_16_FeN_8_
D	22% CNT + 1% Chit + 1% C_32_H_16_FeN_8_

**Table 2:** LOD for different sensors in different buffer solutions and pH. All sensors in Table 2 were used for NO_2_ detection in solutions with either BR or PBS electrolytes. In the Selectivity column are presented the interferent ions.

Sensors (Electrode)	Medium/pH	Linear Range (µM)	LOD (µM)	Selectivity	Reference
AuNPs/MWCNTs/CPE	BR/4.0	0.05–250	0.01	Na^+^, K^+^, Mg^2+^, Ba ^2+^, Ca^2+^, Zn^2+^, Cl^−^, NO_3_^−^, SO_4_^2−^, CO_3_^2−^, ClO_4_^−^, PO_4_^3−^, F^−^	[8]
PdNPs/MWCNTs/GCE	PBS */7.0	0.05–2887	0.022	Ni^2+^, Ca^2+^, Mg^2+^, Fe^2+^, Zn^2+^, Co^2+^, Na^+^, Cl^−^, SO_4_^2−^, SO_3_^2−^, NO_3_^−^, CO_3_^2−^, F^−^	[9]
Ag/Cu/MWCNTs/GCE	PBS/7.0	1–1000	0.2	Cl^−^, NO_3_^−^, SO_4_^2−^, K^+^, Ca^2+^	[10]
Fe(III)P/MWCNTs/GCE	PBS/4.0	1–600; 600–1600	0.5	NaCl, KCl, CaCl_2_, MgSO_4_, ZnCl_2_, Al(NO_3_^−^)_3_, Zn(NO_3_^−^)_2_, K_2_CO_3_, MnCO_3_, Mn(CH_3_COO)_2_, ZnCO_3_, glucose,α-lactose	[11]
Chit-AgNPs/MWCNTPE	BR/4.0	0.1–100	0.03	Br^−^, PO_4_^3−^, SO_4_^2−^, S_2_^−^, I^−^, CH_3_COO^−^, Cl^−^, NO_3_^−^	[14]
Chit/CNs/MWCNTs/GCE	PBS/5.5	5–1000	0.89	Na_2_SO_4_, CH_3_COOK, CaCl_2_, NH_4_NO_3_, CaI, CuSO_4_, glucose, citric acid, ascorbic acid	[15]
P_2_Mo_17_V-PSS-CNTs/Pt-Chit/ITO	PBS/6.0	0.25–4167	0.9	C_2_H_5_OH, Na_2_SO_4_, KbrO_3_, Na_2_CO_3_, KCl, KNO_3_, glucose, acestic acid, citric acid	[16]
AuNPs/Chit/Ti_3_C_2_T_x_/GCE	PBS/7.0	0.5–335; 335–3355	0.069	NH_4_Cl, K_2_SO_4_, NaNO_3_, Na_2_SO_3_, K_2_CO_3_, Cu(NO_3_)_2_	[17]
AuNPs/T i_3_C_2_T_x_/ERGO/GCE	PBS/7.0	0.5–80; 80–780	0.15; 0.051	NH_4_Cl, K_2_SO_4_, NaNO_3_, K_2_SO_4_, K_2_HPO_4_, KCl, Cu(NO_3_)_2_	[18]
ssDNA/SWCNTs/GCE	PBS/4.0	0.6–540	0.15	NaCl, K_2_SO_4_, Ca(NO_3_)_2_, glucose H_2_O_2_, Na_2_SO_3_, ascorbic acid, uric acid, KIO_3_, KI	[19]
Cu^2+^/DNA-SWCNTs/GCE	PBS/3.0	0.03–2600	0.03	Na^+^, K^+^, Mg^2+^, Zn^2+^, Cu^2+^, Cl^−^, F^−^, NO_3_^−^, CH_3_COO^−^, C_2_O_4_^2−^, CO_3_^2−^, PO_4_^3−^	[20]
CNT/Chitosan/C_32_H_16_FeN_8_	BR/4.0	0.4–10	0.12	NO^3−^, SO4^2−^, S_2_O_3_^2^, PO_4_^3−^, CH_3_COO^−^, Cl^−^	This work

* PBS = phosphate buffer solution.

**Table 3:** Determination of nitrite level in water from a well with a C-SPE coated by MAPLE with CNT-Chit-FePc composite.

Added (μM)	Found (μM)	Recovery (%)	RSD (%)
1	1.15	115.7	4.42
3	3.36	112.1	3.98
5	5.19	103.9	3.67
9	8.89	98.8	2.31

## Data Availability

Not applicable.

## Supplementary Materials

The following supporting information can be downloaded at: https://www.mdpi.com/article/10.3390/nano12071138/s1, Figure S1. AFM image of Sample C taken at the edge of the thin layer. Figure S2. (a) FTIR spectra of powder chitosan (as-received from Sigma Aldrich) [31,32,51,52]; Figure S2. (b) FTIR spectra of C_32_H_16_FeN_8_ (as-received from Sigma Aldrich) [33]; Figure S2. (c) FTIR spectra of CNTs (as-received from Sigma Aldrich) [34,35,36,53,54,55]; Figure S3. AFM images of the samples A, B, C and D. Figure S4. EDS spectrum of the MAPLE processed coatings. Figure S5. SEM images of the CNT-Chit-FePc coatings obtained on Si(100) by MAPLE from targets with different compositions: (a) 15% CNT + 1% Chit + 1% FePc in H_2_O (sample A); (b) 15% CNT + 1% Chit + 0.4% FePc in H_2_O (sample B); (c) 22% CNT + 1% Chit + 0.4% FePc) in H_2_O (sample C) and (d) 22% CNT + 1% Chit + 1% FePc in H_2_O (sample D).
