# Supplementary material for: Nitrites Detection with Sensors Processed via Matrix-Assisted Pulsed Laser Evaporation
Source: Nanomaterials (Basel). 2022 Mar 29;12(7):1138. doi: 10.3390/nano12071138 (PMC9000718; doi:10.3390/nano12071138)
Supplement: Supplementary file 1 [file nanomaterials-12-01138-s001.zip › nanomaterials-1612934-supplementary.pdf]

nanomaterials

MDPI

Supplementary Materials

Nitrites Detection with Sensors Processed via Matrix-Assisted Pulsed Laser Evaporation

Cristina Craciun [1,2,†], Florin Andrei [1,3,†], Anca Bonciu [1,2], Simona Brajnicov [1], Tatiana Tozar [1], Mihaela Filipescu [1], Alexandra Palla-Papavlu [1,*] and Maria Dinescu [1]

1 National Institute for Laser, Plasma and Radiation Physics, Lasers Department, 077125 Magurele, Romania; cristina.craciun@inflpr.ro (C.C.); florin.andrei@inflpr.ro (F.A.); anca.bonciu@inflpr.ro (A.B.); brajnicov.simona@inflpr.ro (S.B.); tatiana.alexandru@inflpr.ro (T.T.); mihaela.filipescu@inflpr.ro (M.F.); maria.dinescu@inflpr.ro (M.D.)
2 Faculty of Physics, University of Bucharest, 077125 Magurele, Romania
3 Faculty of Chemistry, University of Bucharest, 030018 Bucharest, Romania
* Correspondence: alexandra.papavlu@inflpr.ro
† These authors contributed equally to this work.

Citation: Craciun, C.; Andrei, F.; Bonciu, A.; Brajnicov, S.; Tozar, T.; Filipescu, M.; Palla-Papavlu, A.; Dinescu, M. Nitrites Detection with Sensors Processed via Matrix-Assisted Pulsed Laser Evaporation. *Nanomaterials* **2022**, *12*, 1138. https://doi.org/10.3390/nano12071138

Academic Editor: Cosimino Malitesta

Received: 10 February 2022
Accepted: 24 March 2022
Published: 29 March 2022

Publisher's Note: MDPI stays neutral with regard to jurisdictional claims in published maps and institutional affiliations.

Copyright: © 2022 by the authors. Submitted for possible open access publication under the terms and conditions of the Creative Commons Attribution (CC BY) license (https://creativecommons.org/licenses/by/4.0/).

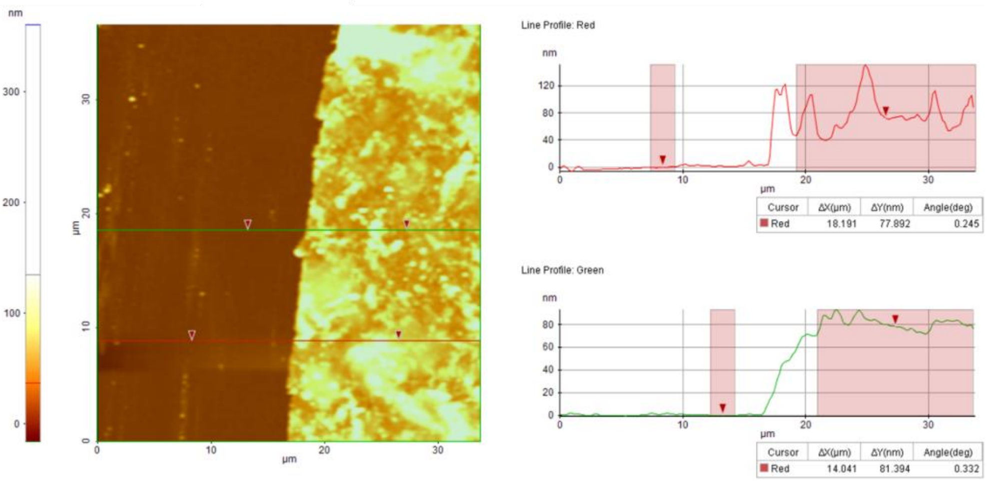

Figure S1. AFM image of Sample C taken at the edge of the thin layer.

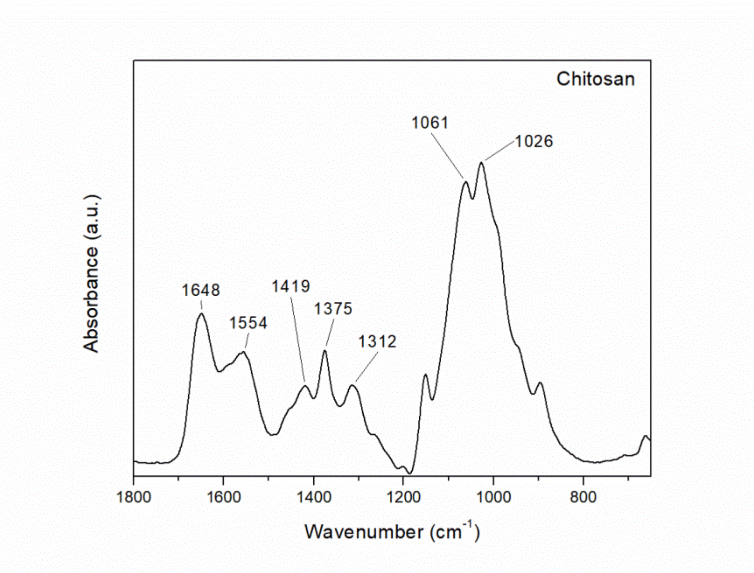

Figure S2. (**a**) FTIR spectra of powder chitosan (as-received from Sigma Aldrich).

The powder chitosan (Figure S2a) shows a strong band with peaks at 3352 and 3292 cm^{-1} that is assigned to N–H and O–H stretching vibrations. The C–H symmetric and asymmetric stretching vibrations are responsible for the absorption bands at 2921 and 2869 cm^{-1}. The presence of residual N-acetyl groups is confirmed due to the presence of bands at 1648 cm^{-1} (C=O stretching vibration of amide I), at 1554 cm^{-1} (N–H bending vibration of amide II), and at 1312 cm^{-1} (C–N stretching vibrations of amide III) [1]. The bands at 1419 and 1375 cm^{-1} correspond to CH_2 bending vibration and CH_3 symmetrical deformations vibration, respectively. The bands at 1061 and 1026 cm^{-1} are assigned to the C–O stretching vibration [2–4].

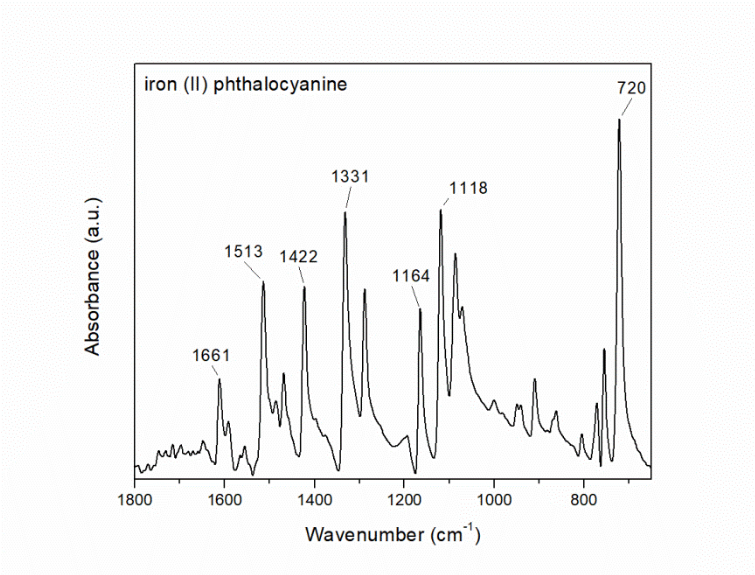

Figure S2. (**b**) FTIR spectra of $C_{32}H_{16}FeN_8$ (as-received from Sigma Aldrich).

The IR spectrum of $C_{32}H_{16}FeN_8$ (Figure S2b) acquired in the range 1650-1000 cm^{-1} presented the characteristic vibration of the phthalocyanine cycle. Therefore, the band at 1661 cm^{-1} represents the C=C stretching vibration, at 1513 and 1422 cm^{-1} the C–C and C–H stretching vibrations, at 1331 cm^{-1} the C–N stretching vibration, at 1164 and 1118 cm^{-1} the C–N stretching vibration, and the band at 720 cm^{-1} the C–N bending vibration [5].

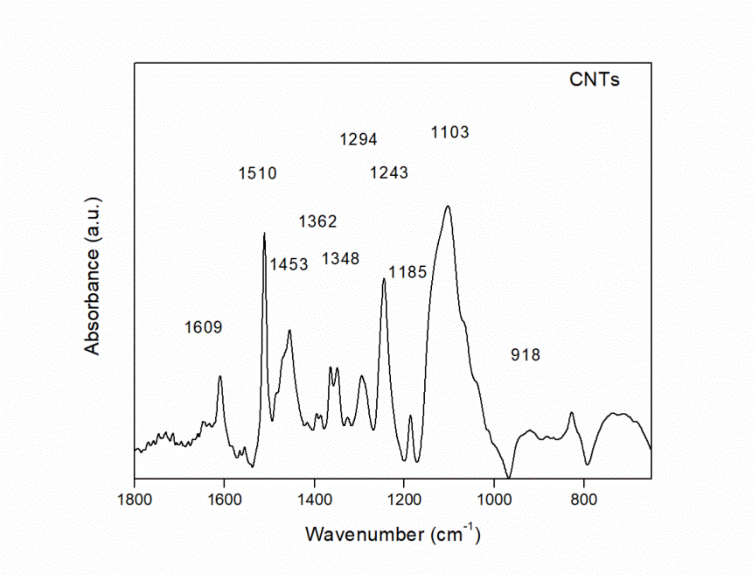

Figure S2. (**c**) FTIR spectra of CNTs (as-received from Sigma Aldrich).

CNTs' infra-red active modes are extremely difficult to detect. Indeed, CNTs lack a static dipole moment, and IR activity is related to a weak dynamic dipole moment. Even so, infra-red spectroscopy has shown great promise in the study of CNT chemistry [6,7]. IR studies on commercial CNT reveal intense bands at 1110, 1535, and 1700 cm^{-1}, as well as a weak band near 830 cm^{-1} [8]. The IR spectrum of the CNT used in this study (Figure S1c) shows the bands at: 3481, 2949, 1609, 1510, 1453, 1362, 1348, 1292, 1243, 1185, 1102, and 918 cm^{-1}. The bands at 3481 and 2949 cm^{-1} represent the O-H stretching vibration (carboxyl groups and –OH shifts in adsorbed water) and C–H_n stretching vibration (CH_2 and CH_3 alkyl moieties), respectively. The O–H vibration is related to amorphous carbon that forms a bond with the atmospheric air [8]. The −C=O stretching vibration associated with carboxylated CNT is observed at 1609 cm^{-1} [9]. The C=C and C–C stretching vibrations that depend on the CNT symmetry can be observed in the range 1580-1500 cm^{-1} and 1200-1100 cm^{-1}. In the spectral range 1300-900 cm^{-1}, the bands are assigned to C–H bending vibrations and combinations of OH deformation and of C–O stretching vibrations. Additionally, the band at 1453 cm^{-1} represents the overlapping of CH_2/CH_3 groups stretching vibrations with OH in-plane deformations of adsorbed water [10]. Finally, from the IR spectra we deduce that the CNTs have functional groups (–OH, –CH, C–O–C, –C=O), implying that they have surface reactivity [11].

The morphology of the films is studied using an AFM – XE 100 from Park System which has a maximum area for scanning of 50 µm × 50 µm and a minimum area of 500 nm × 500 nm. The topography was recorded in non-contact mode and surface roughness of the film (root mean squared) was calculated for a scanning area of 40 µm × 40 µm. The roughness values vary in the range 38 nm – 61 nm for the B, C and D samples and is higher for sample A (~260 nm). In figure S3, the 2D and 3D images of the samples scanned on an area of 5 µm × 5 µm are presented. The worm-like and rod-like structures present on the surface correspond to the CNT and $C_{32}H_{16}FeN_8$, and are also confirmed by the SEM investigations.

Sample A

Sample B

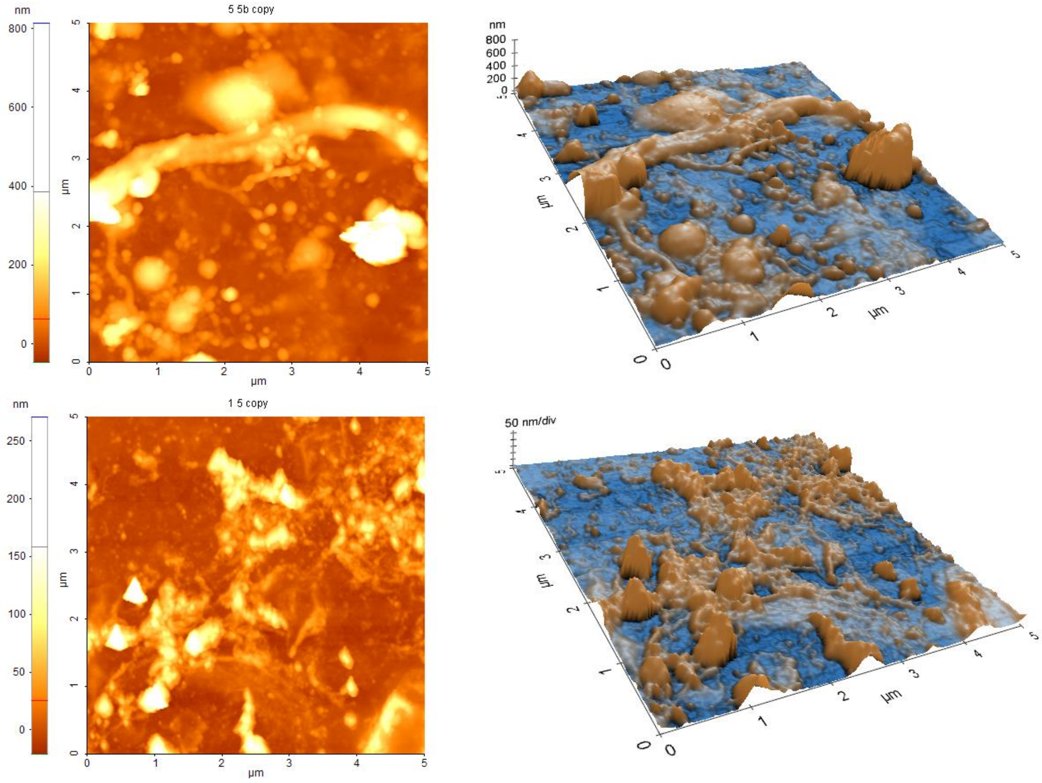

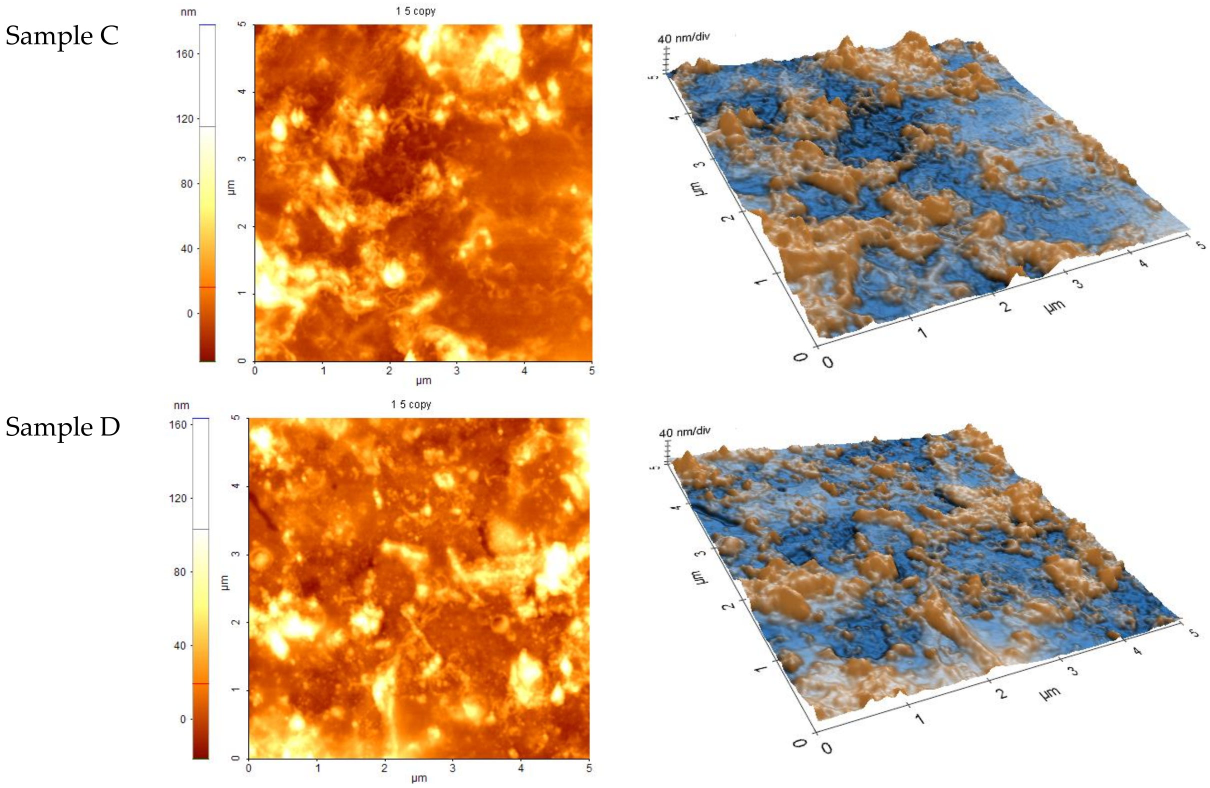

Figure S3. AFM images of the samples A, B, C and D.

An EDX (Element 2CB) attached to a FEI Inspect S SEM operating at 5 keV is used to characterize the elemental composition of obtained films qualitatively; at least three runs were compiled. The samples' elemental compositions as recorded by EDS confirmed that all samples contained C, N, O, and Fe (Figure S4), consistent with drop-casted CNT (C, O), FePc (C, N, O, Fe) and Chitosan (C, N, O). The peak of Si from the EDS spectrum (Figure S4) is characteristic of the substrate used for deposition, and the peak of Au is the result of the 10 nm sputter-coated gold for obtaining electrical conductivity in the SEM analysis. The data are obtained from three different locations on each surface and showed that all of the coatings are uniformly formed on the substrate.

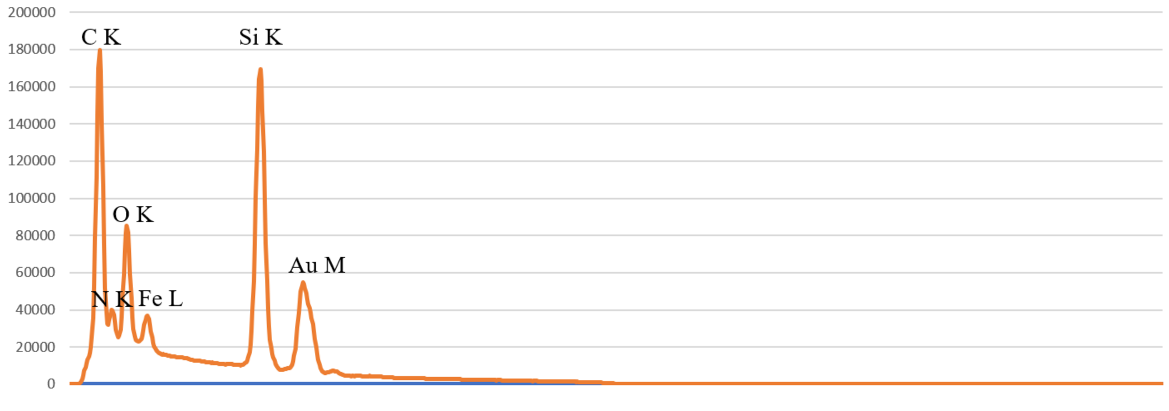

Figure S4. EDS spectrum of the MAPLE processed coatings.

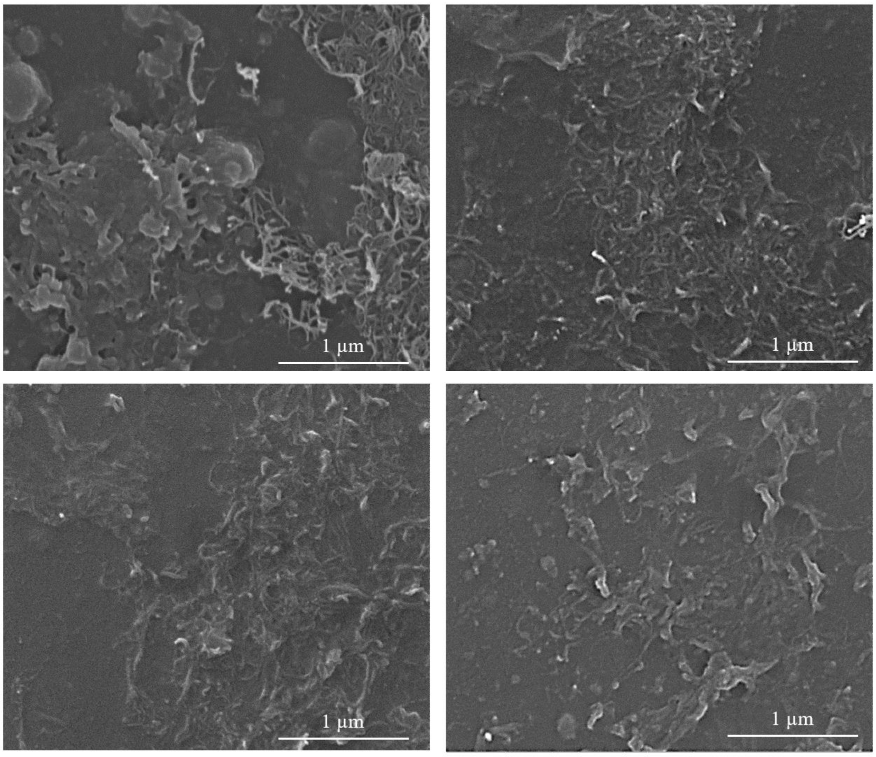

Figure S5. SEM images of the CNT-Chit-FePc coatings obtained on Si (100) by MAPLE from targets with different compositions: (**a**) 15% CNT + 1% Chit + 1% FePc in H_2O (sample A); (**b**) 15% CNT + 1% Chit + 0.4% FePc in H_2O (sample B); (**c**) 22% CNT + 1% Chit + 0.4% FePc) in H_2O (sample C) and (**d**) 22% CNT + 1% Chit + 1% FePc in H_2O (sample D).

References

1. L im, S.-H.; Hudson, S.M. Synthesis and Antimicrobial Activity of a Water-Soluble Chitosan Derivative with a Fiber-Reactive Group. *Carbohydrate Research* **2004**, *339*, 313–319, doi:10.1016/j.carres.2003.10.024.
2. Fernandes Queiroz, M.; Melo, K.R.T.; Sabry, D.A.; Sassaki, G.L.; Rocha, H.A.O. Does the Use of Chitosan Contribute to Oxalate Kidney Stone Formation? *Mar Drugs* **2014**, *13*, 141–158, doi:10.3390/md13010141.
3. Shanmugam, A.; Kathiresan, K.; Nayak, L. Preparation, Characterization and Antibacterial Activity of Chitosan and Phosphorylated Chitosan from Cuttlebone of Sepia Kobiensis (Hoyle, 1885). *Biotechnology Reports* **2016**, *9*, 25–30, doi:10.1016/j.btre.2015.10.007.
4. Song, C.; Yu, H.; Zhang, M.; Yang, Y.; Zhang, G. Physicochemical Properties and Antioxidant Activity of Chitosan from the Blowfly Chrysomya Megacephala Larvae. *Int J Biol Macromol* **2013**, *60*, 347–354, doi:10.1016/j.ijbiomac.2013.05.039
5. Neamtu, M.; Nadejde, C.; Brinza, L.; Dragos, O.; Gherghel, D.; Paul, A. Iron Phthalocyanine-Sensitized Magnetic Catalysts for BPA Photodegradation. *Sci Rep* **2020**, *10*, 5376, doi:10.1038/s41598-020-61980-6.
6. Kim, U.J.; Furtado, C.A.; Liu, X.; Chen, G.; Eklund, P.C. Raman and IR Spectroscopy of Chemically Processed Single-Walled Carbon Nanotubes. *J. Am. Chem. Soc.* **2005**, *127*, 15437–15445, doi:10.1021/ja052951o.
7. Bantignies, J.-L.; Sauvajol, J.-L.; Rahmani, A.; Flahaut, E. Infrared-Active Phonons in Carbon Nanotubes. *Physical Review B* **2006**, *74*, doi:10.1103/PhysRevB.74.195425.
8. Branca, C.; Frusteri, F.; Magazù, V.; Mangione, A. Characterization of Carbon Nanotubes by TEM and Infrared Spectroscopy. *J. Phys. Chem. B* **2004**, *108*, 3469–3473, doi:10.1021/jp0372183.
9. Hussain, S.; Jha, P.; Chouksey, A.; Raman, R.; Islam, S.S.; Islam, T.; Choudhary, P.K. Spectroscopic Investigation of Modified Single Wall Carbon Nanotube (SWCNT). *Journal of Modern Physics* **2011**, *2*, 538–543, doi:10.4236/jmp.2011.26063.
10. Jain, S.M.; Cesano, F.; Scarano, D.; Edvinsson, T. Resonance Raman and IR Spectroscopy of Aligned Carbon Nanotube Arrays with Extremely Narrow Diameters Prepared with Molecular Catalysts on Steel Substrates. *Phys. Chem. Chem. Phys.* **2017**, *19*, 30667–30674, doi:10.1039/C7CP06973A.
11. Ţucureanu, V.; Matei, A.; Avram, A.M. FTIR Spectroscopy for Carbon Family Study. *Critical Reviews in Analytical Chemistry* **2016**, *46*, 502–520, doi:10.1080/10408347.2016.1157013.
